# A phase II trial of TIP (paclitaxel, ifosfamide and cisplatin) given as second-line (post-BEP) salvage chemotherapy for patients with metastatic germ cell cancer: a medical research council trial

**DOI:** 10.1038/sj.bjc.6602682

**Published:** 2005-07-05

**Authors:** G M Mead, M H Cullen, R Huddart, P Harper, G J S Rustin, P A Cook, S P Stenning, M Mason

**Affiliations:** 1 Medical Oncology Unit, C Level West Wing, Southampton General Hospital, Southampton SO16 6YD, UK; 2 University Hospital Birmingham NHS Foundation Trust, Birmingham B15 2TH, UK; 3 Royal Marsden Hospital, Sutton SM2 5PT, UK; 4 Guy's Hospital, London SE1 9RT, UK; 5 Mt Vernon Hospital, Harrow HA6 2RN, UK; 6 MRC Clinical Trials Unit, London NW1 2DA, UK; 7 Velindre Hospital, Cardiff CF4 7XL, UK

**Keywords:** cisplatin, ifosfamide, metastatic germ cell cancer, paclitaxel, salvage chemotherapy

## Abstract

This phase II trial describes the use of TIP chemotherapy (paclitaxel, ifosfamide and cisplatin) as salvage for patients with metastatic germ cell cancer (GCC) who have failed initial BEP (bleomycin, etoposide and cisplatin) chemotherapy. Patients with first relapse following BEP for metastatic GCC, confirmed by biopsy or sequentially rising markers, received four courses of TIP (paclitaxel 175 mg m^−2^ day 1, followed on days 1–5 by ifosfamide 1 g m^−2^ intravenously (i.v.) and cisplatin 20 mg^2^ i.v.) at 3-weekly intervals. The primary outcome measure was response to TIP. In all, 51 patients were registered, of whom 43 were eligible for response assessment. Eight achieved complete remission (CR) and 18 a partial remission with negative markers (PR^−ve^); favourable response rate (FRR=CR+PR^−ve^) 60%, 95% CI (44–75%); survival at 1 year was 70% (56–84%) and failure-free survival 36% (22–50%). In the group of 26 patients meeting the ‘good-risk’ criteria described by the Memorial Hospital, the FRR was 73% (52–88%) compared with 41% (18–67%) for the 17 ‘poor-risk’ patients. These results are inferior to those previously reported for TIP in a single-centre study when it was given more intensively, at higher dose and with growth factor support. Nonetheless, TIP as described here can cure a substantial proportion of patients.

Approximately 85% of patients with newly diagnosed metastatic germ cell cancer (GCC) will be cured when treated with a cisplatin-containing regimen±surgery; BEP (bleomycin etoposide and cisplatin) is currently the worldwide standard treatment. The remaining 15% of patients require salvage treatment and are a relatively heterogeneous group. A proportion may be salvaged surgically, predominantly patients with recurrent mature teratoma or with a late relapse (Baniel *et al*, 1995; Gerl *et al*, 1997). However, most will require chemotherapy.

Two treatment approaches have been used in this population: standard-dose chemotherapy given alone, or standard dose in combination with high-dose chemotherapy plus autologous stem cell rescue. A preliminary analysis of a single randomised trial comparing these approaches has been reported and has shown no difference in survival (Rosti *et al*, 2002).

A variety of prognostic factors determine outlook in these patients (Motzer *et al*, 1991; Gerl *et al*, 1995; Miller *et al*, 1997; Loehrer *et al*, 1998; Fossa *et al*, 1999). The Memorial Hospital Group (MSKCC) divided their population into two groups with a good or poor prognosis (Motzer *et al*, 1991, 2000a, 2000b; Donadio *et al*, 2003). The former group, defined by the presence of a testicular primary, ⩽6 cycles of cisplatin-containing chemotherapy and initial complete response or partial response with normal tumour markers, was evaluated in a phase II study of TIP (paclitaxel, ifosfamide and cisplatin (Motzer *et al*, 2000b). Initial and updated (Donadio *et al*, 2003) results suggest high efficacy for this treatment approach in this group of patients.

The multi-institution study described here has evaluated TIP given at modified dose in a salvage setting to all prognostic groups and has compared the results with the MSKCC salvage chemotherapy experience.

## PATIENTS AND METHODS

### Eligibility

Male patients with the following characteristics were eligible: (i) first relapse after previous BEP chemotherapy given for metastatic GCC, (ii) either sequentially rising serum markers (AFP and/or HCG) or biopsy-proven and unresectable GCC; (iii) age 16–65 years; (iv) ECOG performance status 0–2; (v) glomerular filtration rate of ⩾50 ml h^−1^ and (vi) no evidence of brain metastases.

### Patient registration

Following informed consent, patients were registered by telephoning the MRC Clinical Trials Unit (CTU), London prior to starting treatment. Exceptionally, patients could be registered during the first cycle of TIP if urgent treatment was required and weekends or public holidays prevented earlier registration. The treatment protocol was approved by both national and local Research Ethics Committees.

### Treatment

TIP comprised paclitaxel 175 mg m^−2^ given intravenously (i.v.) over 3 h after appropriate premedication on day 1, followed on days 1–5 by ifosfamide 1 g m^−2^ i.v. over 1 h with mesna 500 mg m^−2^. Following this infusion, a further 500 mg m^−2^ of mesna was given in 1 l of normal saline over 8 h as part of cisplatin hydration. Patients also received cisplatin 20 mg m^−2^ i.v. on days 1–5 with appropriate pre- and postcisplatin hydration.

TIP was recommenced on day 22 using the following dose reduction schedule for ifosfamide and paclitaxel: total WBC >2.0 × 10^9^ l^−1^ and platelets >100 × 10^9^ l^−1^ or WBC>3.0 × 10^9^ l^−1^ and platelets 75–100 × 10^9^ l^−1^; full doses of all drugs. Total WBC >1.5 × 10^9^ l^−1^ and platelets 50–100 × 10^9^ l^−1^; 75% dose ifosfamide and paclitaxel. Total WBC 1.5–2 × 10^9^ l^−1^ and platelets 50–75 × 10^9^ l^−1^; 50% dose ifosfamide and paclitaxel.

Patients with lower blood counts had chemotherapy deferred for 3 days and were treated according to the above schedule on recovery of counts. No dose modifications were made based on previous treatment cycles. Granulocyte colony-stimulating factor (G-CSF) was given at the discretion of the investigator, but was recommended for all future cycles following an episode of neutropenic sepsis.

Cisplatin was given at full dose unless the creatinine clearance fell below 40 ml min^−1^, in which case it was discontinued. If the creatinine clearance recovered subsequently to above this level, cisplatin was initially recommenced at 75% dose. Routine supportive care was offered for neutropenic fever and thrombocytopenia according to the protocols then in use in each institution.

### Clinical evaluation

All patients initially underwent full physical examination together with assessment of the tumour markers AFP, HCG and LDH. Chest X-ray and CT scans of the head, chest, abdomen and pelvis were performed together with routine biochemistry and a 24-h creatinine clearance (or EDTA). During treatment, the AFP and HCG levels were monitored together with chest X-rays. At the completion of chemotherapy, a new CT scan was performed. Marker negative patients with residual resectable masses were assessed surgically and, wherever feasible, these were completely excised.

### Response assessment and toxicity evaluation

All patients had to complete at least one course of chemotherapy before being eligible for response evaluation. Following chemotherapy and/or surgery, the following criteria were defined by the protocol. Complete remission (CR): normal AFP and HCG levels; no radiological evidence of residual tumour masses or, if surgery was performed, complete excision of mature teratoma or necrotic/fibrotic tissue. Incomplete response (IR): persistent elevation of tumour markers or viable cancer seen in surgically resected specimens (including those which were completely resected). Treatment failure: rising tumour markers or radiological progression during chemotherapy. Partial remission, marker negative; (PR^−ve^): normal tumour markers at completion of chemotherapy but nonresectable/resected residual tumour masses. Additionally, the category CR(S) has been used to identify IR patients with no evidence of disease following complete resection of viable malignancy. Failure-free survival (FFS) events were defined as follows: IR/failure of TIP at response assessment (time of failure was taken to be day 1) excluding CR(S) patients; relapse after CR, CR(S) or PR^−ve^ (time of failure was taken to be date of relapse confirmation); and death from any cause (time of failure was date of death, if no prior FFS event observed). Patients without an FFS event were censored at the last assessment date, or start of consolidation chemotherapy (*n*=2). Survival was measured from the date of initiation of chemotherapy to date of death or date last seen. Toxicity was evaluated using the NCIC Common Toxicity Criteria (v2).

### Consolidation therapy and follow-up

In this multicentre study, a number of group members reserved the right to consolidate the treatment results attained with TIP by use of either high-dose chemotherapy with stem cell rescue or involved-field radiotherapy (this latter approach was used predominantly for patients with seminoma and residual masses). High-dose therapy was used less frequently as the study progressed.

On completion of treatment, it was recommended that patients be seen every 2 months during the first year, every 3 months in year 2, every 4 months in year 3, every 6 months in year 4 and annually thereafter.

### Statistical considerations

The primary outcome measure was CR rate after four cycles of TIP. The study was originally designed to accrue, in a single-stage design, 25 patients assessable for response allowing the CR rate to be estimated with a standard error of ⩽10%. A CR rate of ⩾60% with acceptable toxicity was regarded as a suitable target for use of this combination in further studies. After this study commenced, the MSKCC Group published a study using the same drugs, although at different dose, in their good prognosis subgroup (Motzer *et al*, 2000b; Donadio *et al*, 2003). With approval from the independent Trial Steering Committee, we continued our study until we had accrued 25 patients matching these favourable prognosis criteria to see if we could achieve comparable results.

Response rates, FFS and overall survival rates are given with 95% confidence intervals. FFS and overall survival rates are presented as Kaplan–Meier curves.

## RESULTS

### Patient characteristics

In all, 51 male patients from 14 UK centres were registered between April 1998 and October 2002. Initial accrual was slow as the trial was confined to patients relapsing after initial induction with BEP within MRC trial TE20 (de Wit *et al*, 2001), and only later expanded to include any patient relapsing after BEP. Of the 51 patients, 43 were entered after this change, in July 2000.

Eight of the 51 patients (16%) were excluded from the primary analysis. Seven were ineligible: four had normal markers with no biopsy confirmation of active cancer, one had brain metastases, one was treated as adjuvant following complete resection of active cancer and one was registered too late (during the third cycle of chemotherapy)). In addition, one patient developed an anaphylactic reaction to paclitaxel in the first treatment cycle and was thereby withdrawn from the study, and inevaluable for response.

All the following results relate to the 43 eligible patients (26 good prognosis (Motzer *et al*, 2000b), 17 poor prognosis (Motzer *et al*, 2000a)) whose characteristics are shown in Table 1. The population comprised four patients with early relapse (entering this study ⩽2 months after the start of their last cycle of BEP), nine with late relapse (⩾2 years from completion of BEP) and the remaining 30 patients with intermediate relapse. Histology was nonseminoma in 33 (77%), and 37 (86%) had a gonadal primary.

### Chemotherapy delivery

In all, 36 patients (84%) received all four cycles of TIP, five patients received three cycles and one patient two cycles. The final patient received five cycles of TIP, the final cycle being given in error. The median (range) time between treatment cycles 1–2, 2–3 and 3–4, respectively, were 21 days (19–33), 21 days (19–28) and 21 days (15–49). The proportion of patients receiving >85% of the individual drugs were 80.5% for cisplatin, 78.1% for ifosfamide and 78.1% for paclitaxel. The median (range) relative dose intensity (actual daily dose intensity divided by planned, full dose, dose intensity over four courses of treatment) was 0.97 (0.58–1.02) for cisplatin, 1.03 (0.58–0.95) for ifosfamide and 0.95 (0.63–1.03) for paclitaxel.

### Toxicity

One toxic death occurred following the third TIP cycle, the patient dying from staphylococcal septicaemia while neutropenic. Grade 3 or 4 leucopenia was recorded in 64% of patients, neutropenia in 70% of patients and thrombocytopenia in 35% of patients; 12 patients (28%) had granulocytopenic fever (with neutrophils <1.0 × 10^9^ l^−1^).

### Response rates, FFS and survival

Eight patients (19%) achieved CR, seven from TIP alone, one from TIP and complete resection of a necrotic mass. In total, 18 (42%) achieved PR^−ve^ (one had resection of one mass with others observed). Five patients (12%) had CR(S) (of whom two went on to receive adjuvant etoposide). Eight patients (19%%) had IR (of whom two underwent partial resections and one a complete resection of one of several metastatic sites) and three (7%) treatment failure/early death (one of whom had complete resection of one of several metastatic sites). The favourable response rate as defined by the protocol (FRR_p_=CR+PR^−ve^) was 60%, 95% CI (44–75%). Including CR(S) patients in the favourable response category (FRR_c_), the FRR_c_=72% (56–85%).

The corresponding figures according to MSKCC risk group are shown in Table 2; the overall FRR_p_ was 73% (52–88%) for the good-risk patients and 41% (18–67%) for the poor-risk group. The FRR_p_ (95% CI) for the three groups early (*n*=4), intermediate (*n*=30) and late relapse (*n*=9) were, respectively, 0% (0–60%), 67% (47–83%) and 67% (30–93%).

Adjunctive treatment was given to seven patients (six of whom were in the favourable prognosis group). Four (three seminoma, one mixed tumour) achieved PR^−ve^ status and received radiotherapy to residual masses; one achieved PR^−ve^ status but proceeded to high-dose therapy with stem cell support; one patient had an IR (complete resection of viable tumour) and was given adjuvant oral etoposide and finally one patient had an IR (partial resection of viable tumour) and had further high-dose chemotherapy. All seven patients remain alive and progression free.

In all, 28 FFS events have occurred comprising one early toxic death, 11 IR/failure to TIP (nine have died, one is alive with active disease, one is alive and disease free), four relapses after CR(S) (three have died, one is alive with active disease) and 12 relapses after CR or PR^−ve^ (five have died, four are alive with active disease, three are alive and disease free). Thus, 19 patients are alive and disease free, six are alive with active disease and 18 have died. Of the four patients currently disease free following an FFS event, salvage therapy was high-dose chemotherapy (two patients), gemcitabine+cisplatin (one patient) and surgery+radiotherapy (one patient). The median follow-up time of the 25 survivors is 26 months (range 11–70 months).

**Figure 1:**
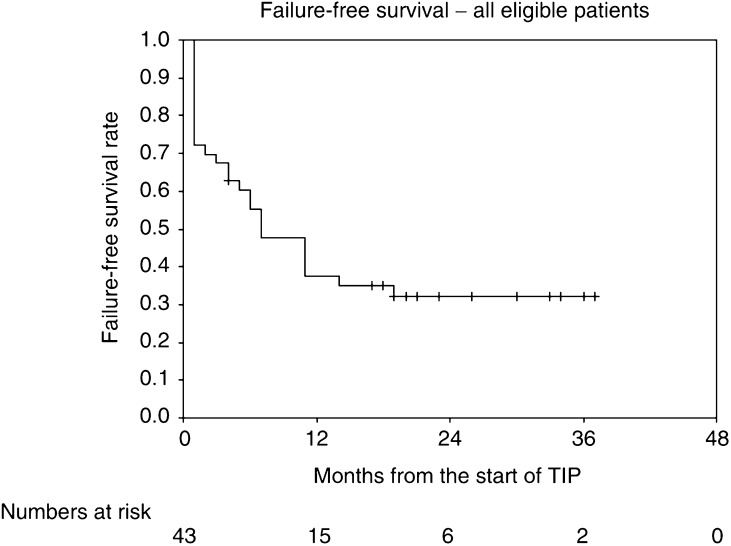
Failure-free survival, all eligible patients.

**Figure 2:**
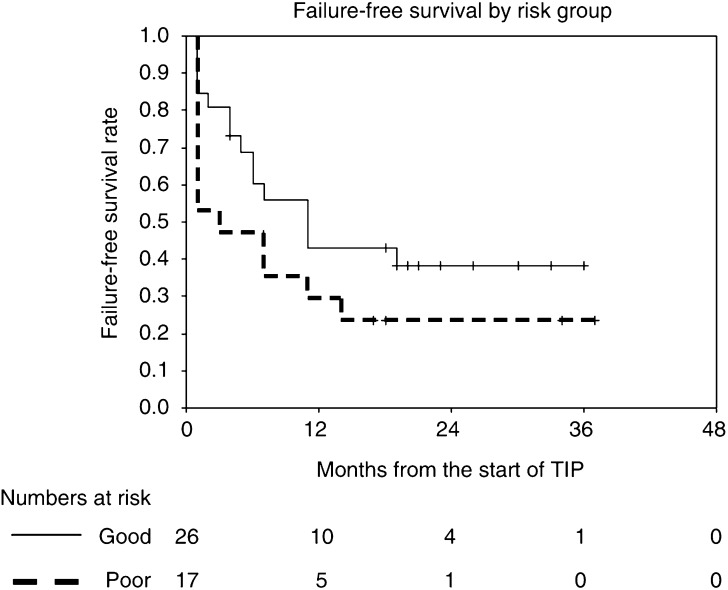
Failure-free survival by risk group.

**Figure 3:**
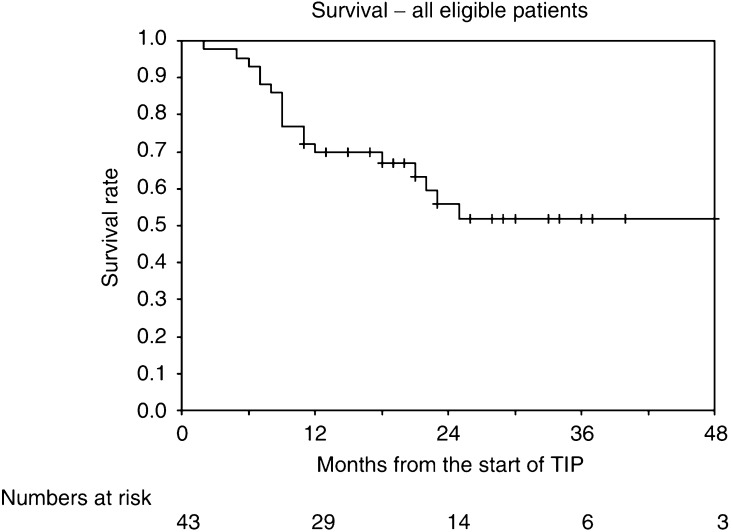
Survival, all eligible patients.

**Figure 4:**
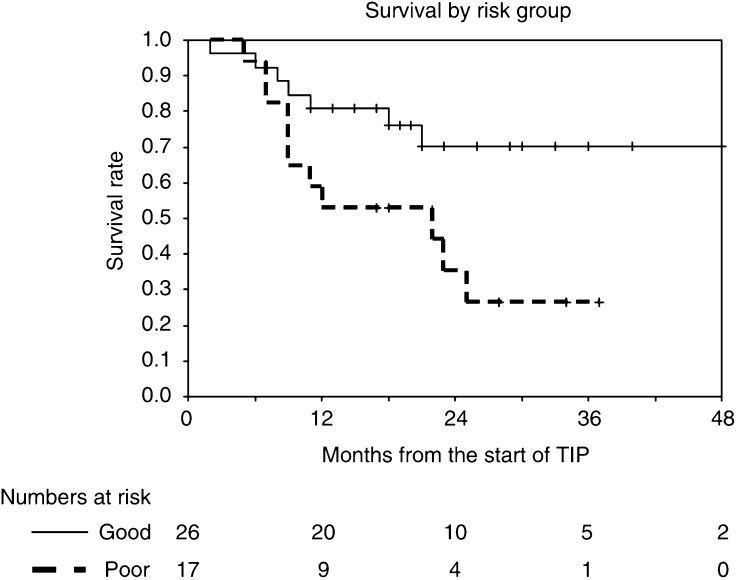
Survival by risk group.

Overall FFS and FFS by MSKCC risk groups are shown in Table 2 and Figures 1 and 2. The overall FFS rate at 1 year is 38% (95% CI 23–53%); the corresponding rates for the good- and poor-risk subgroups are 43% (23–63%) and 29% (8–51%), respectively. Overall survival and survival by MSKCC risk groups are shown in Figures 3 and 4. The overall survival rate at 1 year is 70% (56–84%) and the 1-year survival rates for the good- and poor-risk subgroups are 81% (64–98%) and 53% (29–77%), respectively.

Of the nine seminoma patients (five good risk, four poor risk), six are currently alive and disease free (three received additional therapy with radiotherapy and one with high-dose chemotherapy as adjunctive therapy). Of 33 patients with NSGCT, 13 are alive and disease free.

## DISCUSSION

Patients with metastatic GCC developing first progression of active cancer despite initial cisplatin-containing chemotherapy almost all require salvage combination chemotherapy. The exceptions are patients developing late (>2 years) relapse with resectable disease (Baniel *et al*, 1995; Gerl *et al*, 1997).

Salvage chemotherapy should be given with curative intent and published series suggest 20–57% of patients may achieve long-term disease-free status with this and subsequent salvage therapy (Motzer *et al*, 1991, 2000a, 2000b; Pizzocaro *et al*, 1992; Miller *et al*, 1997; Bhatia *et al*, 2000). Prognostic factors associated with an improved outlook include gonadal primary site (Motzer *et al*, 1991; Pizzocaro *et al*, 1992; McCaffrey *et al*, 1997; Loehrer *et al*, 1998), achievement of initial CR (or PR^−ve^ status) with first-line therapy (Motzer *et al*, 1991; Pizzocaro *et al*, 1992; Gerl *et al*, 1995; McCaffrey *et al*, 1997; Loehrer *et al*, 1998; Fossa *et al*, 1999), time to relapse (Baniel *et al*, 1995; Gerl *et al*, 1995, 1997; Fossa *et al*, 1999), low serum markers at relapse (Motzer *et al*, 1991; Fossa *et al*, 1999) and (probably) histology, with seminoma favourable (Miller *et al*, 1997). These factors vary greatly in published salvage series, rendering comparison between regimens impracticable.

Most patients retain cisplatin sensitivity at relapse, and this drug has remained a cornerstone of salvage regimens. In early series, cisplatin was combined with vinblastine (or etoposide) and ifosfamide (VeIP, VIP; Pizzocaro *et al*, 1992; Farhat *et al*, 1996; McCaffrey *et al*, 1997; Miller *et al*, 1997; Loehrer *et al*, 1998), and numerous series have reported cure rates of 15–42% with these combinations used alone or with surgery in patients with NSGCT (Pizzocaro *et al*, 1992; McCaffrey *et al*, 1997; Loehrer *et al*, 1998).

The obvious need to improve these results has yielded two different strategies: the increasing use of new drugs with paclitaxel (Motzer *et al*, 1994; Bokemeyer *et al*, 1996; Sandler *et al*, 1998), gemcitabine (Bokemeyer *et al*, 1999; Einhorn *et al*, 1999), oxaliplatin (Kollmannsberger *et al*, 2002a), irinotecan (Kollmannsberger *et al*, 2002b; Miki *et al*, 2002) and epirubicin (Madani *et al*, 2003) currently under evaluation as single agents or in combination (Hinton *et al*, 2002; Miki *et al*, 2002; Kollmannsberger *et al*, 2004), and the development of high-dose chemotherapy, particularly since the advent of autologous stem cell rescue and growth factor support (Motzer *et al*, 1992, 2000a; Beyer *et al*, 1997; Bhatia *et al*, 2000; Rosti *et al*, 2002; Kollmannsberger *et al*, 2004).

The initial approach to salvage chemotherapy has varied from centre to centre, although the increasing safety of high-dose treatment has led to a number of centres, including Indiana, recommending this as initial salvage treatment. This has the advantage of an increased likelihood of achieving cure compared with third-line or later use of high-dose therapy (Rick *et al*, 2001), but the disadvantage of poor tolerance of subsequent salvage chemotherapy for the 40–50% of patients destined to fail this treatment approach (Pont *et al*, 1997; Beyer *et al*, 2002). It also represents overtreatment for the 50–60% of good prognosis patients who would have been cured with standard-dose treatment (Fossa *et al*, 1999).

One, two or multiple cycles of high-dose therapy using a variety of two or three drug regimens have been used in a phase II setting. However, a single randomised trial (IT94) under the auspices of the European Bone Marrow Transplant Group has provided sobering data (Rosti *et al*, 2002). A total of 280 patients with cisplatin-sensitive disease were randomised to four cycles of VIP/VeIP or three cycles of this treatment followed by high-dose chemotherapy with carbopec (high-dose carboplatin, etoposide and cyclophosphamide) with stem cell rescue. The 1-year event-free survival and 3-year survival (52% in both arms) were essentially identical.

The alternative approach is standard-dose chemotherapy incorporating newer drugs. The most commonly evaluated regimen is TIP. The MSKCC used this regimen in 30 patients (Motzer *et al*, 2000b) – now updated to 46 patients (Donadio *et al*, 2003) – in a good prognosis group but at higher doses than those reported here (paclitaxel 250 mg m^−2^ by 24-h infusion, ifosfamide 6 gm m^−2^ in divided dose) and at higher intensity, as no dose reductions were made and G-CSF was routinely administered. Whereas all patients in the MRC study had failed BEP, the previous treatment given to the MSKCC patients was more heterogeneous and was carboplatin based in two cases.

The MSKCC results were superior when compared with those described here in a broadly comparable, good-risk group with longer follow-up. In the updated study report published to date only in abstract form (Donadio *et al*, 2003), 46 patients received TIP (41 nonseminoma, five seminoma), 16 in the setting of late relapse. In all, 32 patients achieved CR (70%) and two PR^−ve^, with a total favourable response rate of 74%. Three patients relapsed and with a median follow-up of 52 months, 78% remain alive. The increased CR rate, and markedly reduced PR^−ve^ rate presumably reflect a greater use of postchemotherapy surgery, although only three patients are recorded as having viable malignancy resected to achieve CR in the updated series.

TIP has also been evaluated by a German group (Porcu *et al*, 2000). A totoal of 80 patients failing initial or salvage cisplatin-based combination chemotherapy were treated with three cycles of TIP (doses as used by MRC, but ifosfamide 1.2 g m^−2^ i.v. daily × 5) with G-CSF support. It was planned that all patients would proceed to high-dose chemotherapy using carboplatin, etoposide and thiotepa. The response rate to TIP was 11% CR and 29% PR^−ve^ (total 40%). In all, 78% of patients proceeded to high-dose therapy. With a median follow-up of 3 years, 21 patients (26%) were failure free with 22 alive (26%). This was a more heavily pretreated population than ours. Nonetheless, this seems a disappointing result.

The MSKCC Group have published separate data on the salvage therapy of patients relapsing with a poor prognosis. In total, 37 patients were treated with two cycles of paclitaxel and ifosfamide at 2-week intervals and accompanied by stem cell harvest. The patients were then treated with three cycles of high-dose carboplatin and etoposide at 14–21 day intervals with stem cell support. Despite the highly adverse prognostic features of this group, 41% remained failure free at a median follow-up time of 2½ years. Our results using TIP in a comparable group, with much shorter follow-up, again appeared inferior.

A number of publications are now available describing experience with single agents (Motzer *et al*, 1994; Bokemeyer *et al*, 1996, 1999; Sandler *et al*, 1998; Einhorn *et al*, 1999; Kollmannsberger *et al*, 2002a, 2002b) or drug combinations (Miki *et al*, 2002; Hinton *et al*, 2002; Madani *et al*, 2003; Kollmannsberger *et al*, 2004) in patients with end-stage and cisplatin-refractory disease. Paclitaxel, gemcitabine and oxaliplatin have been evaluated as single agents, and the combinations paclitaxel and gemcitabine (Hinton *et al*, 2002), oxaliplatin and gemcitabine (Kollmannsberger *et al*, 2004), cisplatin and irinotecan (Miki *et al*, 2002) and cisplatin and epirubicin (Madani *et al*, 2003) as combinations. Comparison of these regimens is not possible because of the heterogeneous nature of the groups treated. However, the achievement of ongoing complete remission in a proportion of cases receiving all four described drug combinations is certainly a provocative finding.

How should contemporary patients relapsing after first-line chemotherapy be treated? There is no definite answer. The single randomised trial showing no benefit to high-dose treatment has been criticised as only one cycle of high-dose treatment was used. Patients in a good prognosis group can still reasonably be treated with standard-dose salvage regimens. Further prospective randomised trials are indicated but would clearly need to be international. Entry of these patients into prospective studies wherever possible is indicated.

In conclusion, our multicentre experience of TIP chemotherapy demonstrates that a substantial proportion of patients, particularly those in the MSKCC favourable group, can achieve long-term FFS. The results of patients with early relapse or those failing to respond satisfactorily to initial BEP are poor and alternative treatment approaches are clearly required. In a future study, we plan to intensify TIP, firstly by avoiding dose reductions by use of growth factors and secondly through the addition of gemcitabine.

## Figures and Tables

**Table 1:** Patient characteristics at study entry (*n*=43)

	**Number of patients**	**%**
*Primary site*		
Testis	37	86.00
Mediastinal	3	7.00
Retroperitoneal	1	2.30
Not known	2	4.70
		
*Age (years)*		
Median	34	
Minimum	20	
Maximum	51	
		
*Histology*		
Seminoma	9	20.90
Nonseminoma	33	76.70
Not known (high HCG, no biopsy)	1	2.30
		
*Sites*		
Abdominal	24	55.80
Mediastinal	9	20.90
Supraclavicular	6	14.00
Lung	14	32.60
Pleura	2	4.70
Mesenteric	2	4.70
Markers only	3	7.00
Liver	1	2.30
Bone	1	2.30
Kidney	1	2.30
Inguinal nodes	1	2.30
Pelvis	1	2.30
		
*BHCG (IU l^−1^)*		
Median	28	
Minimum	Normal	
Maximum	9944	
		
*AFP (KU l^−1^)*		
Median	4	
Minimum	Normal	
Maximum	89 000	
		
*LDH (IU l^−1^)*		
Median	391	
Minimum	133	
Maximum	4534	
		
*Relapse interval*		
<2 months	4	9.30
2 months to 2 years	30	69.80
>2 years	9	20.90

**Table 2:** Response rates, FFS and overall survival

	**Response (*N*, %)**								
**Group**	**CR**	**PR MK-ve**	**Complete resection of viable malignancy CR(S)**	**IR**	**Treatment failure/early death**	**Favourable (CR+PR) response rate (FFR_p_) (95% CI)**	**Favourable (CR+PR+CR(S)) response rate (FFR_c_) (95% CI)**	**1-year FFS rate (95% CI)**	**1-year overall survival rate (95% CI)**
All patients	8 (19%)	18 (42%)	5 (12%)	8 (19%)	4 (9%)	60% (44–75)	72% (56–85)	38% (23–53)	70% (56–84)
MSKCC good risk	7 (27%)	12 (46%)	2 (8%)	3 (12%)	2 (8%)	73% (52–88)	81% (61–93)	43% (23–63)	81% (64–98)
MSKCC poor risk	1 (6%)	6 (35%)	3 (18%)	5 (29%)	2 (12%)	41% (18–67)	59% (33–82)	29% (8–51)	53% (29–77)

CR=complete response; PR=partial response; IR=incomplete response; FFS=failure-free survival; 95% CI=95% confidence interval.

## Appendix A1

This study was initiated by the Medical Research Council (now National Cancer Research Institute) Testicular Cancer Group. The principal investigators were Professor M Mason (Cardiff) and Dr GM Mead (Southampton). The trial was coordinated by the MRC Clinical Trials Unit, London (formerly the MRC Cancer Trials Office, Cambridge, UK); the senior Clinical Trial Manager was Pat Cook and the trial statistician Sally Stenning. The following centres and clinicians entered patients into the trial:

Southampton General Hospital/Royal South Hants Hospital (10): GM Mead, P Simmonds.
Birmingham Oncology Centre (9): MH Cullen.
Royal Marsden Hospital (7): R Huddart, DP Dearnaley.
Guy's Hospital (5): P Harper.
Cookridge Hospital, Leeds (5): WG Jones (retired).
Mt Vernon Hospital (5): GJS Rustin.
Bristol Oncology Centre (2): JD Graham.
Beatson Oncology Centre, Glasgow (2): P Vasey.
Velindre Hospital, Cardiff (1): J Barber, M Mason.
Aberdeen Royal Infirmary (2): D Bissett, A Hutcheon.
Leicester Royal Infirmary (1): FJ Madden (retired).
Lincoln County Hospital (1): T Sreenivasa.
Walsgrave Hospital, Coventry (1): A Stockdale.
Christie Hospital, Manchester (1): PM Wilkinson.
